# Biology, Pathobiology and Gene Therapy of CNG Channel-Related Retinopathies

**DOI:** 10.3390/biomedicines11020269

**Published:** 2023-01-19

**Authors:** Maximilian J. Gerhardt, Siegfried G. Priglinger, Martin Biel, Stylianos Michalakis

**Affiliations:** 1 Department of Ophthalmology, University Hospital, LMU Munich, Mathildenstraße 8, 80336 Munich, Germany; 2 Department of Pharmacy—Center for Drug Research, Ludwig-Maximilians-Universität München, 81377 Munich, Germany

**Keywords:** achromatopsia, CNG, cyclic nucleotide-gated channel, cGMP, channelopathies, Ca^2+^, gene therapy, inherited retinal disease, IRD, knockout, photoreceptor, vision, retinitis pigmentosa, RP

## Abstract

The visual process begins with the absorption of photons by photopigments of cone and rod photoreceptors in the retina. In this process, the signal is first amplified by a cyclic guanosine monophosphate (cGMP)-based signaling cascade and then converted into an electrical signal by cyclic nucleotide-gated (CNG) channels. CNG channels are purely ligand-gated channels whose activity can be controlled by cGMP, which induces a depolarizing Na^+^/Ca^2+^ current upon binding to the channel. Structurally, CNG channels belong to the superfamily of pore-loop cation channels and share structural similarities with hyperpolarization-activated cyclic nucleotide (HCN) and voltage-gated potassium (KCN) channels. Cone and rod photoreceptors express distinct CNG channels encoded by homologous genes. Mutations in the genes encoding the rod CNG channel (*CNGA1* and *CNGB1*) result in retinitis-pigmentosa-type blindness. Mutations in the genes encoding the cone CNG channel (*CNGA3* and *CNGB3*) lead to achromatopsia. Here, we review the molecular properties of CNG channels and describe their physiological and pathophysiological roles in the retina. Moreover, we summarize recent activities in the field of gene therapy aimed at developing the first gene therapies for CNG channelopathies.

## 1. Introduction

Cyclic nucleotides, such as cAMP and cGMP, are second messengers that regulate important signaling pathways in our body by controlling the activity of several effector proteins, including cyclic nucleotide-binding domain (CNBD)-containing cation channels. Among CNBD-containing ion channels, cyclic nucleotide-gated (CNG) channels are the only strictly ligand-gated channels because their opening requires binding of cAMP or cGMP [1]. In vertebrates, the CNG channel gene family includes six homologous members. *CNGA1*, *CNGA2*, and *CNGA3* encode subunits that confer key channel properties and have been shown to form functional homotetrameric ion channels in heterologous expression systems [1]. *CNGA4*, *CNGB1,* and *CNGB3* encode structurally similar subunits that cannot form functional ion channels by themselves, but are important for the correct localization of native channel complexes and confer specific biophysical properties to the channel [1]. Four of the CNG channel genes are linked to inherited retinal disorders (IRD): mutations in *CNGA1* and *CNGB1* are known to cause retinitis pigmentosa (RP) and mutations in *CNGA3* and *CNGB3* cause achromatopsia (ACHM).

## 2. Insights on Structure and Activation of CNG Channels

**Figure 1:**
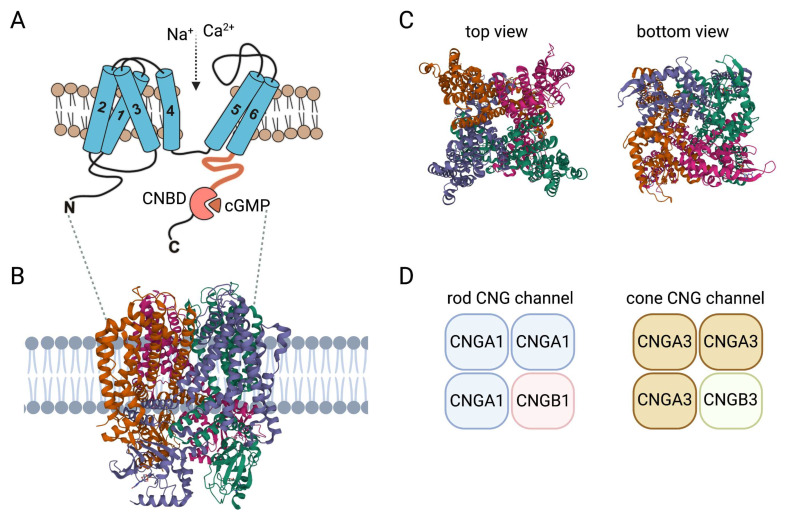
Structure and activation of CNG channels. (**A**) Membrane topology of CNG channel subunits: 1–6, transmembrane segment 1–6; C, carboxy-terminus; CNBD, cyclic nucleotide-binding domain; N, amino-terminus. (**B**) Model of the CNG channel complex embedded in the plasma membrane based on the human rod CNGA1/CNGB1 channel structure (PDB 7RHH). (**C**) Top and bottom views of the heterotetrameric human rod CNGA1/CNGB1 channel complex. (**D**) Subunit composition of the CNG channels from rods and cones. Structures in this figure were generated with the RSCB PDB 3D View tool (www.rcsb.org/3d-view/ accessed on 19 Dec 2022) based on PDB 7RHH.

Each of the six CNG channel genes encode a membrane protein with six α-helical transmembrane segments (S1–S6), a channel core consisting of a reentrant pore (P) loop between S5 and S6, and cytosolic N- and C-termini. S5, S6, and the intervening reentrant pore (P) loop form the actual pore domain [2] (Figure 1). Similar to classical voltage-gated channels, S4 hosts multiple positively charged residues and S1–S4 form a voltage-sensor-like domain (VSLD). However, unlike canonical voltage-gated channels, the VSLD structure is segmented and the positively charged amino acids are not regularly spaced [3]. This hinders appropriate charge movement and helps to explain why the function of the CNG channel does not depend on voltage [3]. The C-terminus harbors the CNBD and is connected to the S6 via the C-linker (Figure 1). Single particle cryo-electron microscopy structures of native human rod and cone CNG channels confirmed the heterotetrameric 3:1 stoichiometry of the native rod [4] and cone [5] channel complex previously postulated on the basis of biophysical and biochemical experiments [6,7,8,9] (Figure 1). The native CNG channels in the outer segments of rod photoreceptors are heterotetramers consisting of three CNGA1 and one CNGB1 subunit. The CNG channel in the outer segments of cone photoreceptors is formed by three CNGA3 and one CNGB3 subunit. Unlike other members of the voltage-gated channel superfamily, but similar to HCN channels, the four subunits of the tetrameric CNG channel complex are arranged in a non-swapped configuration where the VSLD interacts only with the pore domain of the same subunit [3,10].

CNG channel structures captured at different activation states in the presence of cGMP and/or pharmacological blockers revealed details about the architecture of the ion-conducting pore and contributed to our understanding of channel function and the effects of pathogenic mutations on channel function [4,5,11,12,13,14]. Based on comparisons of available structures in the open and closed states and previous mutagenesis studies [15,16,17,18], activation of the CNG channel is thought to involve coordinated movements of at least three basic elements, the CNBD, the C-linker with its gating ring, and the channel gate [10,19]. The binding of cyclic nucleotides to the CNBD results in a rotational change of the entire C-terminus relative to the pore. The C-linker, a domain that allosterically couples the binding of cyclic nucleotides to the channel gate via its gating ring, also follows this rotation and moves partially upward. The channel gate, located in the intracellular part of the S6 segment, is constricted and presumably kept in a closed state by the constant forces of the C-linker. After binding of the ligand, the movements described above cause the inhibitory forces of the C-linker to subside and the channel pore is opened, allowing ions to permeate. Despite a sequence identity of only about 35%, the structures of CNGB1 and CNGA1 align well and exhibit similar domain arrangements, which results in a quite symmetrical pore of the closed heterotetrameric CNGA1/CNGB1 channel [4]. In homotetrameric CNG channels, all four A subunits show symmetrical rotational movements that lead to pore opening. However, opening of the heterotetrameric CNGA1/CNGB1 channel is asymmetrical [4]. Only the two CNGA1 subunits left and opposite of the CNGB1 subunit show the movements known from the homomeric channel, whereas CNGB1 and the other CNGA1 subunit barely move, resulting in an asymmetrical open-pore geometry [4]. Structures of the heteromeric cone CNG channel in different activation states are still missing. However, the solved structures revealed an asymmetrical pore architecture already at the closed state with an arginine residue of the CNGB1 S6 projecting directly into and occluding the ion conduction pathway of the pore [5,14]. Mutation of this arginine to a glycine led to a higher single channel conductance [4]. Despite these significant advances in our understanding of the structure and function of CNG channels, there are still gaps in our knowledge, particularly with respect to the gating mechanism of the cone CNG channel or the N- and C-terminal regions, which, for technical reasons, could so far only be modeled for the bovine rod CNG channel [4,5,14]. Further insights are expected from studies aimed at determining the structure of specific pathogenic ACHM- or RP-causing CNG channel mutants with yet unexplained effects on channel structure and function.

## 3. Role of CNG Channels in Signal Transduction in Photoreceptors

**Figure 2:**
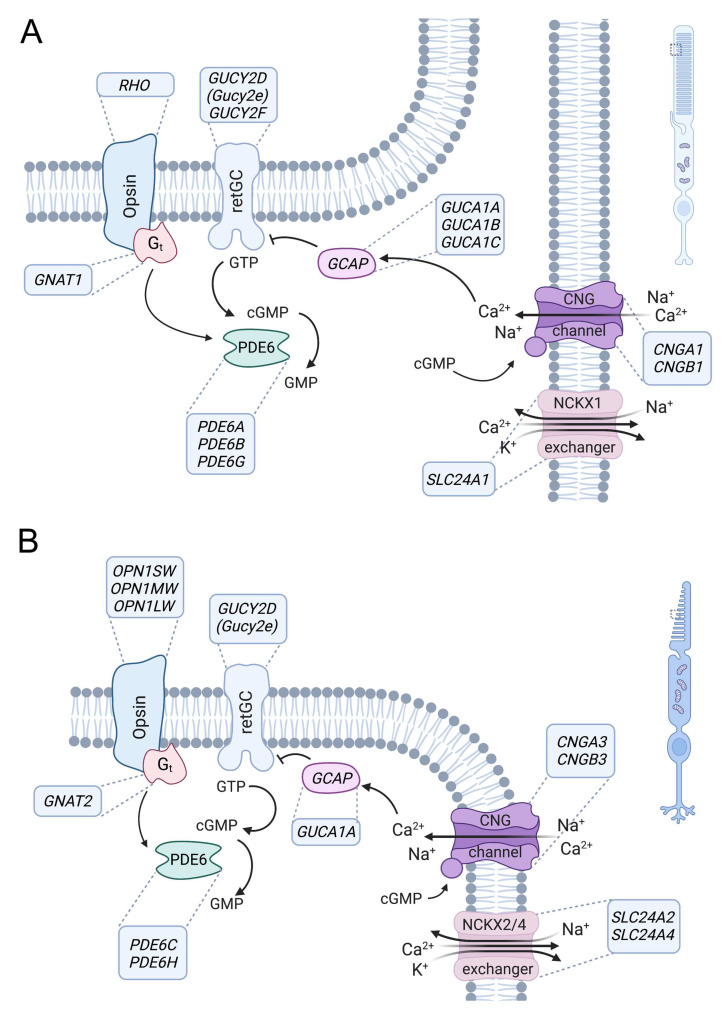
Role of CNG channels in rod and cone photoreceptor signaling. Phototransduction in outer segments of rod (**A**) and cone (**B**) photoreceptors. The principle of the phototransduction is similar in both cell types, but key proteins are encoded by distinct but homologous genes (human gene names are indicated in the boxes next to the proteins). In the dark, the cyclic nucleotide-gated (CNG) channel (CNGA1/B1 in rods and CNGA3/B3 in cones) of the outer membrane is kept open by high concentrations of cyclic guanosine monophosphate (cGMP) produced by retinal guanylyl cyclase (retGC) (note: GUCY2E is a pseudogene in humans, whereas Gucy2e is functional in rodents and the major retGC encoding gene). The resulting influx of Na^+^ and Ca^2+^ depolarizes the plasma membrane. Light activates the opsin, which in turn activates transducin (Gt), whose alpha subunit activates a phosphodiesterase (PDE6) that leads to hydrolysis of cGMP. The decrease in the cGMP concentration leads to the closure of the CNG channel, resulting in membrane hyperpolarization. Ca^2+^ is an important regulator of phototransduction. At high concentrations, Ca^2+^ binds to guanylyl-cyclase-activating proteins (GCAP), leading to the inhibition of retGC. High Ca^2+^ concentrations also lead to a slight reduction in the cGMP affinity of the CNG channel through Ca^2+^/calmodulin-mediated feedback inhibition (not illustrated). Ca^2+^ is cleared from the outer segment via a Na^+^-Ca^2+^-K^+^-exchanger (NCKX1 in rods, NCKX2/4 in cones). At low Ca^2+^ levels, Ca^2+^-free GCAPs can bind and activate retGC to stimulate cGMP production and reopen the CNG channel.

Vertebrates have two types of highly specialized photoreceptors, rods and cones, which have similar but distinct phototransduction signaling cascades and enable the detection of light under different ambient conditions (Figure 2). Rods mediate vision in low light, whereas daylight vision is conferred by cones and only to a lesser extent by rods. The cone visual system also enables color vision because it can discriminate between wavelengths by comparing inputs from two (in most vertebrates) or three (in humans and some nonhuman primates) types of cones, which are equipped with different cone opsin variants with varying spectral sensitivities [20,21]. In both rods and cones, signal transduction follows the same principle and is facilitated by enzymes that control the concentration of cyclic guanosine monophosphate (cGMP). In turn, cGMP controls activation of the CNG channel in the plasma membrane of outer segments (Figure 2). In the dark, the constant activity of transmembrane guanylyl cyclases results in high cGMP concentrations that maintain the CNG channels in an open conformation [22,23]. CNG channels conduct a constant, non-inactivating Na^+^ and Ca^2+^ current (“dark current”) that depolarizes the photoreceptor and promotes glutamate release at the photoreceptor synaptic terminals. In response to a light-triggered conformational change, opsins, being G-protein-coupled receptors, release their G protein transducin, which in turn binds to and activates PDE6-type cGMP phosphodiesterases [24]. PDE6 enzymes hydrolyze cGMP, leading to CNG channel closure and photoreceptor hyperpolarization, thus reducing synaptic glutamate release. Ca^2+^ influx into the outer segments is mediated exclusively by the CNG channels [1,25,26] and balanced by Ca^2+^ outflow via the Na^+^/Ca^2+^, K^+^ exchangers [25,27,28,29,30]. The closure of CNG channels upon light stimulation, together with the constant activity of Na^+^/Ca^2+^, K^+^ exchangers, leads to a decrease in intracellular Ca^2+^ concentration. This reduced Ca^2+^ contributes to recovery from the light response by modulating the activities of PDE6 and guanylyl cyclases [24,31,32,33].

The key activities in this phototransduction cascade are the same in rods and cones but are often mediated by functionally equivalent proteins encoded by distinct genes. This is also the case for the rod and cone CNG channels, which have basically the same functional properties and differ only in some specific features. Notable examples are the higher Ca^2+^ permeability of the cone CNG channel and the stronger Ca^2+^-dependent inhibition of ligand sensitivity in the rod CNG channel [1,34]. However, these differences cannot fully explain the different sensitivity and kinetics of rods and cones [1,35].

## 4. Genetics and Biology of the Rod and Cone CNG Channel

### 4.1. The Rod CNGA1/CNGB1 Channel

Dysfunction of the rod CNG channel causes autosomal recessive retinitis pigmentosa (RP) [36,37]. RP comprises a genetically diverse group of progressive degenerative retinal diseases primarily affecting the photoreceptors of the retina [38]. Common symptoms of RP include night blindness, progressive constriction of the visual field, and abnormal migration and accumulation of pigment in the retina [39]. The disease is characterized by a primary loss of rod function followed by degeneration and loss of rod photoreceptors, which can vary from patient to patient depending on the underlying gene mutation. As rod loss progresses, cone photoreceptor morphology and function become impaired, affecting daylight vision. This leads to a gradual constriction of the visual field under daylight conditions. In advanced stages, RP leads to impaired visual acuity and may lead to blindness in the final stage. More than 70 genes have already been linked to RP, with different forms of inheritance [40] (see also https://web.sph.uth.edu/RetNet/ accessed on 19 December 2022). Many RP genes encode proteins involved in the phototransduction cascade (Figure 2) or proteins required for the maintenance of photoreceptor architecture. *CNGA1* (OMIM #123825) and *CNGB1* (OMIM #600724) are linked to autosomal recessive RP (arRP). The prevalence of mutations that cause *CNGA1*-RP varies in different geographic regions ranging from 1–8% of arRP [37,38,41,42,43,44]. Most of the identified *CNGA1* mutations cause deletions of key functional domains or result in impaired membrane trafficking [1,2,37,45]. Mutations in *CNGB1* account for 1–4% of arRP cases, and again the prevalence is expected to vary in different geographic areas [36,38,40,42,46,47,48,49]. Although the known *CNGB1* mutations cause only minor deletions or single amino acid substitutions, the phenotype is comparable to the RP phenotype in *CNGA1*-RP patients. Functional characterization studies of some *CNGB1* mutations have been performed, showing that some mutations affect rod CNG channel stability or transport, whereas others do not affect expression but result in functionally inactive CNG channels [36,48,50,51].

Today, animal models exist for both *CNGA1*-RP and *CNGB1*-RP [52,53,54,55,56,57,58] (Table 1). However, most phenotypic data come from *Cngb1* animal models, some of which have been available for more than two decades and have been extensively characterized [54,55,56,57,59,60,61,62].

A naturally occurring *Cnga1* mutation has been identified in a Shetland sheepdog breed with progressive retinal atrophy [52]. However, detailed information on the retinal phenotype is still missing. Recently, a mouse model with a targeted deletion in exon 2 of *Cnga1* was reported [53]. *Cnga1* knockout mice carry a 65-bp frame-shift deletion that, although not experimentally verified at the protein level, should lead to a premature stop codon and loss of most of the Cnga1 protein shortly after deletion. Homozygous mice show loss of most photoreceptors at 16 weeks of age [53]. Dark-adapted electroretinogram (ERG) responses to a single flash of 3 cd*s/m^2^ were greatly reduced in these mice after 3 weeks, which further decreased after 10 weeks [53]. More recently, an N-ethyl-N-nitrosourea (ENU)-induced *Cnga1* mutant mouse model was generated and characterized [63]. The mutant mice carry a c.1526 A > G mutation in *Cnga1* that leads to a Y509C exchange in the CNBD of the Cnga1 protein. Y509 corresponds to Y513 in the human CNGA1 protein and participates in the formation of the b3 strand of the CNBD [1]. The Y509C mutation appears to impair the stability of the rod CNG channel complex, resulting in a complete loss of Cnga1 and Cngb1 proteins despite largely unchanged mRNA levels. As a result, rod-driven ERG responses were diminished by 3 weeks of age. Non-functional rods degenerated over time, and from the sixth month of life, secondary progressive degeneration of cones was observed, which was completed by 1 year of age, a time point at which ERG responses were no longer measurable [63].

Nearly two decades ago, a first *Cngb1* knockout mouse model was described [54]. This mouse model carries a deletion of exon 26 of the *Cngb1* gene which encodes S6. The deletion also leads to a reading frame shift that generates a stop codon at the first triplet of exon 27, thus terminating translation [54]. This leads to a loss of Cngb1 protein expression, but also to degradation of the Cnga1 protein, which appears to require Cngb1 for proper expression. These *Cngb1*-X26 knockout mice completely lack rod CNG channel function which manifests in diminished responses of rods to light and reduced scotopic ERG responses. The dysfunction is paralleled by the progressive degeneration of the rods and the secondary degeneration of the primarily unaffected cones. Degeneration of the cone photoreceptors begins at 6 months of age, when approximately 50% of the rods have been lost. At about 1 year of age, only 10–20% of the photoreceptors are left in the retina [54]. Overall, the phenotypes of *Cngb1*- and *Cnga1*-deficient mice are very similar in terms of disease manifestation and progression. This is most likely because the loss of either CNG channel subunit leads to the secondary degradation and loss of the remaining CNG channel subunit protein. In both cases, this results in a complete loss of the rod CNG channel complex and its functions, which explains the phenotypic similarities observed in the corresponding mouse models.

A spontaneous mutation in *CNGB1* was also found in a Papillon dog breed with markedly reduced or absent rod function and slowly progressive retinal degeneration [55]. Interestingly, this c.2387delA;2389_2390insAGCTAC mutation found in this naturally occurring dog model leads to premature termination of the Cngb1 protein at almost the same position as in the engineered *Cngb1*-X26 mice [54,55,58]. Comparative analyses revealed that *CNGB1*-RP patients and mouse and dog models with Cngb1 deficiency have a similar phenotype characterized by early loss of rod function and slow degeneration of rod photoreceptors along with a secondary decrease in cone function [59]. The existence of this canine model for *CNGB1*-RP is of great importance for evaluating the translational potential of future gene therapies. This is due to the structural similarities of canine and human eyes in terms of size and, to some extent, in terms of the spatial distribution of photoreceptor subtypes (e.g., the canine eye has a cone-rich visual stripe that partially mimics the human macula). Moreover, the fact that *CNGB1* mutant dogs and *Cngb1*-X26 mice have genetic alterations that result in the termination of the transcript at an almost identical position increases confidence in extrapolating results from the two models to the situation in humans.

The CNGB1 locus encodes multiple transcripts with distinct expression patterns. The longest transcript uses all 33 exons and produces the CNGB1 (also termed CNGB1a) subunit of the rod CNG channel [64]. The first 11 or 16 exons (including a unique alternative exon) [64] give rise to separate cytosolic proteins corresponding to portions of the glutamic-acid-rich protein (GARP) in the N terminus of CNGB1a. Another mouse model with a genetic modification in exon 1 of *Cngb1* (*Cngb1*-X1 mice) was generated to study the effect of GARP deletion [56]. In principle, *Cngb1*-X1 mice exhibited similar functional defects as *Cngb1*-X26 mice, but they showed more dramatically impaired outer rod segment morphology, suggesting that soluble and channel-bound GARP proteins are essential for rod disc morphogenesis and outer segment integrity. Additional studies have shown that channel-attached and soluble GARPs are inherently unfolded [65] and play distinct roles in shaping the morphology of the outer rod segment, transport, and function of the CNG rod channel [50,61,64,66,67,68,69]. These findings are relevant to *CNGB1*-RP patients who harbor mutations in parts of the *CNGB1* locus that give rise to GARP proteins.

### 4.2. The Cone CNGA3/CNGB3 Channel

Dysfunction of the cone CNG channel causes achromatopsia (ACHM), a rare retinal disease that is inherited in an autosomal recessive manner and affects approximately one in 30,000 individuals [70]. Unlike color blindness, in which mutations in genes encoding the various cone photopigments affect only spectral sensitivity [71], ACHM has severe consequences for all aspects of daylight vision. Symptoms include poor visual acuity, photophobia, nystagmus, and lack of color discrimination [72]. The symptoms reflect a functional defect of the cone photoreceptors that occurs in early infancy and is characterized by a lack of light-adapted ERG but preserved scotopic ERG signal [73,74]. In addition to the functional deficits, structural changes can be observed in the cone-rich central portion of the retina, ranging from loss of cone outer segments to profound atrophy of the retina [72]. Up to 90% of ACHM cases are due to mutations in *CNGA3* (OMIM #216900) and *CNGB3* (OMIM #262300) [75,76,77]. The remaining cases are due to mutations in *ATF6* (OMIM #616517), *GNAT2* (OMIM #613856), *PDE6C* (OMIM #613093), *PDE6H* (OMIM #610024), or yet unknown genes [78,79].

To date, more than 250 mutations in *CNGA3* [80,81,82,83,84,85,86,87,88] and more than 160 mutations in *CNGB3* [76,77,80,83,89,90,91,92,93,94] were found to cause ACHM in humans. Mutations in *CNGB3* are more common in Europe and the United States and account for 50–60% of ACHM cases [76,89], and in the Netherlands even close to 90% [83]. Most *CNGB3* mutations are nonsense, frameshift, or splice mutations [77,80]. A missense mutation in the *CNGB3* gene (S435F) was identified in colorblind individuals originating from the Pingelap atoll of Micronesia [90]. In this small island ACHM is very frequent and affects nearly 10% of the native population [90,91,95,96,97]. An estimated 28–36% of patients in the Western population carry mutations in *CNGA3* (ACHM2) [81,84]. In the Middle East and Chinese populations, mutations in *CNGA3* account for approximately 80% of ACHM cases [42,80,98,99]. Interestingly, a digenic and triallelic inheritance pattern with mutations in both *CNGA3* and *CNGB3* was also found in a subset of ACHM patients [100]. The majority of *CNGA3* mutations are missense mutations affecting only single amino acid residues of the protein [81,82,83,84,85,86,88]. Folding, intracellular processing, and transport are thought to be impaired [101]. While some insights have been gained, the precise mechanisms linking specific amino acid substitutions to the ACHM phenotype are still poorly understood. The effects of individual amino acid substitutions on CNGA3 protein function have been studied largely in vitro [85,100,101,102,103,104,105,106,107,108,109,110,111,112,113,114,115,116].

Several genetically modified and naturally occurring animal models of ACHM exist (Table 1) that have helped to elucidate disease mechanisms and serve as disease models for the preclinical development of emerging gene therapies [58,70]. The first animal model of ACHM described was the *Cnga3* knockout mouse, which carries a homozygous deletion of exon 7, leading to the deletion of all channel domains downstream of the third transmembrane segment (S3), including the pore and the CNBD [117]. This results in a complete loss of cone CNG channel function. As a consequence, *Cnga3* knockout mice show a selective deficiency of cone-mediated light responses from birth [117], followed by the progressive degeneration and cell death of cones [117,118]. Cone degeneration affects M- and S-cones differentially and cell death proceeds significantly faster in ventral and nasal (S-cone-rich) than in dorsal and temporal (M-cone-rich) parts of the retina. Ventral cones are almost completely missing after the third postnatal month, whereas residual dorsal cones are present even in aged knockout mice [118]. In addition, a naturally occurring mouse model of ACHM has been described. The mouse line designated *cpfl5* (cone photoreceptor function loss 5) carries a point mutation in *Cnga3* leading to a p.T203A substitution in the cytoplasmic loop between S2 and S3. To date, no missense mutation affecting this phylogenetically conserved threonine, corresponding to p.T224 in human CNGA3 protein, has been reported. Although the exact mechanism remains unclear, this mutation results in the loss of Cnga3 protein expression and a phenotype similar to that observed in the *Cnga3* knockout mouse [119].

Moreover, a naturally occurring sheep model of ACHM was reported with congenital visual impairment characterized by diminished cone, but normal rod function [120,121]. Affected lambs were found to be homozygous for a nonsense mutation in *Cnga3* (p.R236X) [121]. Subsequently, a *Cnga3* missense mutation (p.G540S) was identified in another breed, causing a similar phenotype of day blindness [120]. In addition, two spontaneous canine models of ACHM have been described [122]. One is a Labrador retriever with a p.V644del mutation that removes a conserved valine within a C-terminal domain shown to be important for heterotetrameric channel assembly and stability [6]. The second model is a German shepherd, carrying a p.R424W mutation. This amino acid is found in the gating ring within the C-linker that connects transmembrane domain S6 with the CNBD and is conserved in eukaryotes. Importantly, a mutation that leads to a R-to-W substitution of the corresponding human *CNGA3* sequence (p.R410W) was also found in achromatopsia patients [82]. Recent cryo-electron microscopy (cryo-EM) studies with the *C. elegans* channel tax-4 version (CNGA3/p.R421W) have shed light on the potential pathogenic mechanism of this missense mutation [11]. Careful analysis of cryo-EM data in conjunction with electrophysiological and biochemical data led to the conclusion that this R-to-W substitution in the gating ring destabilizes the closed state of the channel and favors spontaneous channel opening in the absence of the ligand [11]. Thus, if the channel is expressed in the cone outer segments of CNGA3/p.R421W patients, its excessive activity could induce (e.g., Ca^2+^-mediated) cell death and cone degeneration.

Various animal models also exist for *CNGB3*-linked ACHM (Table 1). A *Cngb3* knockout mouse with a genetic deletion that causes a frame shift and removes part of S1 and all other channel domains has been described [123]. These knockout mice lack *Cngb3* protein expression and show strongly reduced Cnga3 protein levels. The lack of the cone CNG channel severely impairs cone function and leads to progressive cone degeneration reminiscent of the *Cnga3* knockout mouse phenotype [123,124]. Residual cone function is observed in this model, most likely conferred by irregular homomeric CNGA3 channels. In addition to the *Cngb3* knockout mouse, a naturally occurring mouse model designated *cpfl10* has been described [125]. The mice carry a c.692G>A point mutation leading to p.R231H [125]. Basic characterization of the mouse line revealed a loss of cone-driven ERG responses and slow progressive degeneration of the cones [125].

Two naturally occurring canine cone degeneration models with mutations in *Cngb3* have been identified in Alaskan malamute and German shorthaired pointer breeds [126]. Genetic analysis has shown that in the Alaskan malamute, the complete gene is deleted, while in the German shorthaired pointers the disease is caused by a missense mutation c.784G > A;p.D262N affecting a conserved aspartate residue in S2 [126]. Affected Alaskan malamute pups develop day blindness and photophobia resembling the clinical phenotype of human ACHM patients. Symptoms are present only in bright light, while vision in dim light is normal. The cone ERG signals begin to diminish a few weeks after birth and are extinguished in older affected dogs [127]. Recently, a viral vector-delivered CRISPR-Cas9 strategy was used to generate an in situ knockout model of *CNGB3*-ACHM in cynomolgus monkeys [128]. This acute model can provide valuable information about the pathobiology of *CNGB3* deficiency in a non-human primate retina with a foveo-macular structure similar to that in the human eye.

The existence of the numerous animal models of *CNGA3*- and *CNGB3*-ACHM not only contributes to a better understanding of the biology of the cone CNG channel, but also greatly facilitates the development and testing of potential treatments. As mentioned for the rod CNG channel, the availability of the large animal models with morphological similarities to the human eye (in terms of size and cell distribution) is of paramount importance for translational studies. Other than for rod CNG channel models, *Cnga3*- and *Cngb3*-deficient mouse models exhibit some phenotypic differences, possibly due to cone-specific morphologic features that, in the absence of Cngb3, still allow a more efficient transport of homotetrameric Cnga3 channels to the outer segment, thereby supporting residual CNG channel function. However, in the absence of Cnga3, Cngb3 cannot support channel function on its own.

**Table 1:** Overview of retinal CNG genes, associated human diseases, animal models, and preclinical studies. NCT ID, www.clinicaltrials.gov identifier (accessed on 19 December 2022). OMIM, Online Mendelian Inheritance in Man. POC, prove of concept.

Gene	Chromosomal Location	Phenotype, OMIM	Animal Models	POC Studies	Preclinical Safety Studies	Clinical Trials (NCT ID)
*CNGA1*	4q12	RP49, 613756	knockout mouse [53] mutant mouse [63] canine model [52]	-	-	-
*CNGB1*	16q21	RP45, 613767	knockout mouse [54,56] canine model [55,57,59]	Refs. [59,129,130]	-	-
*CNGA3*	2q11.2	ACHM2, 600053	knockout mouse [117] mutant mouse [131] canine model [122] ovine model [121]	Refs. [119,132,133,134,135,136,137,138]	Refs. [137,138,139,140,141,142]	02610582 02935517 03758404 03278873
*CNGB3*	8q21.3	ACHM3, 605080	knockout mouse [123] mutant mouse [125] canine model [126] in situ NHP model [128]	Refs. [143,144,145]	Refs. [146,147,148]	02599922 03001310 03278873

## 5. Gene Therapy for the Treatment of CNG Channelopathies

To date, there is no curative treatment for any CNG channelopathy, and clinical treatment is currently limited to specialized genetic counseling, use of visual aids, and tinted contact lenses or glasses to reduce symptoms of photophobia. Our improved understanding of CNG channel biology, the availability of suitable animal models, and the emergence of efficient and safe adeno-associated virus (AAV) vectors led to the initiation of several gene therapy programs for potential treatment of CNG-channel-related retinopathies (Table 1).

ACHM and RP caused by mutations in CNG channel genes are inherited in an autosomal recessive manner. Unlike most autosomal inherited retinopathies, it is not necessary to remove a (dominant) pathogenic variant, and the addition of a functional gene copy would be sufficient for therapeutic benefit. Therefore, the gene therapy approaches aim at adding a healthy copy of the disease-causing gene into the affected cells (in this case, the cone or rod photoreceptors). Some of these so-called gene supplementation (or augmentation) approaches have already reached the clinical phase of development. The following sections provide an introduction into the AAV vector technology and summarize the key findings of preclinical studies and publicly available data from clinical trials.

### 5.1. The AAV Vector Technology

AAVs are small (diameter of 25 nm), non-enveloped, non-pathogenic DNA viruses that can only replicate in the presence of adeno, papilloma, or herpes viruses. Multiple naturally occurring AAV serotypes exist and have been explored for their use as vectors for gene transfer [149,150]. The AAV vector platform has already been clinically validated, and five gene therapy products have been approved for ophthalmic, CNS, and other indications in recent years (www.ema.europa.eu and www.fda.gov accessed on 19 December 2022). More than three decades ago, AAVs were vectorized by the replacement of the viral genes rep and cap with a gene expression cassette of choice [151]. The AAV rep and cap genes are provided in trans during recombinant AAV production process in form of helper plasmids [152]. AAVs consist of a 60-mer capsid of structural viral proteins (VP1, VP2, and VP3) assembled in a 5:5:50 ratio and an approximately 4.7 kb long single-stranded DNA genome containing the desired gene expression cassette for the gene of interest, flanked by two inverted terminal repeats (ITR) [153]. Such AAV-derived viral vectors can deliver their genome into the target cell nucleus where it remains episomal and transcribes the gene of interest without integrating into the host genome. In recent years, AAV vectors have evolved as the gold standard gene delivery vector for retinal photoreceptors and retinal pigment epithelial cells with proven tropism and efficacy. Their success is based on the fact that they are easy to produce at large scale and show a generally good safety profile with limited immunogenicity and dose-dependent toxicity [154,155].

### 5.2. Gene Therapy for CNG-Channel-Linked RP

To date, there are no gene therapy approaches for *CNGA1*-linked RP. For *CNGB1*-linked RP, successful proof-of-concept studies for AAV-based gene supplementation have been reported in both the *Cngb1* knockout mouse model [129] and the *Cngb1* mutant dog model [59]. To enable the efficient packaging and rod-specific expression of the relatively large full-length *Cngb1* cDNA (~4 kb), the two studies used an AAV expression cassette with a short rod- [129] or photoreceptor-specific [59] promoter to drive expression of a species-matched *Cngb1* cDNA (e.g., mouse or canine). In both species, subretinal injection of therapeutic AAV gene supplementation vectors (serotype 5 or 8) led to efficient expression of the Cngb1 protein and the restoration of CNG channel expression and localization. This resulted in the improvement of rod-mediated retinal function, preservation of retinal structure, and delay of secondary cone degeneration. Finally, treated *Cngb1* knockout mice as well as *CNGB1* mutant dogs performed significantly better than untreated controls in rod-dependent vision-guided behavior tests [59,129]. These promising results facilitated the initiation of translational studies with a humanized vector version (AAV5-RHO-CNGB1) in which a short human rhodopsin promoter drives expression of the full-length human *CNGB1* [130]. When administered via single subretinal injection in 4-week-old Cngb1 knockout mice, AAV5-RHO-CNGB1 led to efficient expression of the human CNGB1 protein in mouse rods and restored the expression of the endogenous mouse Cngb1 protein [130]. The treatment resulted in a dose-dependent recovery of rod-driven ERG responses and the preservation of retinal structure [130]. Studies in large animal models are currently underway to support the implementation of this gene therapy approach for the future treatment of *CNGB1*-RP patients.

### 5.3. Gene Therapy for CNG-Channel-Linked ACHM

#### 5.3.1. ACHM Gene Therapy: Preclinical Proof-of-Concept Studies

An AAV5 vector expressing human *CNGB3* cDNA under control of one of three different truncated versions of the human M/L opsin promoter was evaluated in a gene augmentation approach in dogs affected by achromatopsia due to mutations in *CNGB3* [143]. The dogs were injected unilaterally into the subretinal space at 3 to 81 weeks of age. Improvement in cone function was observed as early as 4 weeks after treatment under photopic conditions using ERG and behavior and persisted for at least 14 months. The best treatment results were achieved in 3-week-old-animals, whereas treatment was minimally effective in dogs 1 year of age and older [143]. The exact reasons for the age-dependence of treatment are not known, but could be related to morphological changes observed in later stages of the disease. Accordingly, efficacy in this dog model was improved in older dogs when the AAV gene augmentation was combined with the administration of ciliary neurotrophic factor (CNTF), which is known to cause a temporal deconstruction of photoreceptor outer segments [144].

A positive proof-of-concept for gene supplementation therapy with an AAV5 vector driving expression of mouse *Cnga3* cDNA under control of a short mouse S opsin promoter was also achieved in the *Cnga3* knockout mouse model of achromatopsia [132]. Two-week-old mice treated with subretinal injection showed cone-driven ERG responses, normalization of cGMP levels and expression of cone CNG channel complexes and opsins, and delay of cone cell death. In addition, ganglion cells from treated but not untreated *Cnga3* knockout mice showed cone-driven light-evoked spiking activity, suggesting that signals generated in the outer retina are transmitted to the brain. Finally, it was demonstrated that the newly acquired sensory information was translated into cone-mediated, vision-guided behavior [132]. The therapeutic effect was stable for at least 12 months and was also seen with an AAV8 serotype vector or with treatment at 3 months of age [133]. Similar effects were obtained in different *Cnga3* mouse models after subretinal administration of an AAV5 vector and the human M/L opsin promoter [119] as well as intravitreal delivery of engineered AAV8 (Y447, 733F) [134] or AAV2.GL [135] vectors. In line with the dog studies, an AAV8 vector driving expression of the human *CNGB3* cDNA under control of a short human *ARR3* promoter was shown to efficiently rescue cone-driven ERG responses and visual acuity in the *Cngb3* knockout mouse model of achromatopsia [145]. Successful AAV-based gene supplementation therapy has also been described in the Awassi sheep model of *CNGA3* achromatopsia [136]. Significant long-term improvement in cone function was demonstrated for at least 6 years after a single dose of an AAV vector expressing human *CNGA3* [137,138].

These promising preclinical studies led to the initiation of a total of five independent gene therapy programs for *CNGA3*- and *CNGB3*-linked achromatopsia. Safety studies in sheep and non-human primates revealed some inflammation after subretinal *CNGA3* gene delivery, but overall showed an acceptable safety profile for at least two different translatable *CNGA3* gene therapy products [138,139,140,156]. Safety data have not yet been published for the third *CNGA3* gene therapy product. For one of the two *CNGB3* programs, safety data obtained in mice, dogs, and cynomolgus monkeys were published showing acceptable safety with vector- and dose-dependent inflammation and toxicity [146,147,148].

#### 5.3.2. ACHM Gene Therapy: Clinical Studies

All of the aforementioned translational programs for *CNGA3*- and *CNGB3*-linked achromatopsia have already reached the clinical phase [70] (Table 1). The German academic research consortium RD-CURE initiated the first clinical trial which evaluated the effect of three different doses (1 × 10^10^, 5 × 10^10^, and 1 × 10^11^ total vector genomes per eye) of AAV8.CNGA3 administered via subretinal injection into one eye. The study enrolled nine patients in three dose groups. Despite the highly invasive delivery procedure, which involved vitrectomy and the detachment of the foveo-macular retina, the treatment was well tolerated and resulted in dose-independent mild and transient procedure- or drug-related adverse events [157,158] and transient subclinical induction of inflammatory markers [140,142]. Treatment led to improvement in secondary end points related to cone function, including improvement in visual acuity and contrast sensitivity from baseline in all treated patients [158], which showed a tendency to be dose-dependent and persisted until at least 3 years after treatment [157]. A phase IIb clinical trial targeting treatment of the second eye of the first patients and treatment of children aged 6 to 12 years is currently ongoing (Table 1).

Four other programs are currently in phase I/II of clinical trials, two on CNGA3-ACHM and an additional two on CNGB3-ACHM (Table 1). Preliminary safety and efficacy data from industry-sponsored clinical trials testing the safety and efficacy of the gene therapy products AGTC-401 and AGTC-402 in CNGB3- and CNGA3-related achromatopsia, respectively, were presented at the annual meeting of the American Society for Vision Research and Ophthalmology (ARVO). Both gene therapy products used a modified AAV2 capsid with three surface-exposed Y to F mutations (Y275F, Y447F, and Y733F), and designated AAV2tYF and a 1.7 kb human M/L opsin promoter driving expression of either *CNGA3* or *CNGB3*. The AGTC-401 study enrolled 21 adult and 10 pediatric CNGB3 achromatopsia patients in six dose groups (1.2 × 10^11^ vector genomes (vg)/mL to 3.2 × 10^12^ vg/mL). The AGTC-402 trial included 16 adult and 8 pediatric *CNGA3* achromatopsia patients distributed into five dose groups (4 × 10^10^ vg/mL to 3.2 × 10^12^ vg/mL). In both studies, dose-limiting toxicity was noted at the highest dose (3.2 × 10^12^ vg/mL) in children, which included uveitis and posterior segment changes. The highest dose was better tolerated in adult patients. Gene therapy improved photosensitivity in some *CNGB3*-ACHM patients but less in *CNGA3*-ACHM patients. Long-term follow-up studies were initiated for both programs (Table 1), but recently, the company announced that the *CNGA3*-ACHM program will not be developed further. The other industry-sponsored CNGA3 and CNGB3 gene therapy programs have released only limited safety data at clinicaltrials.gov (https://clinicaltrials.gov/ct2/show/results/NCT03758404, accessed on 19 December 2022), but communicated plans to initiate late-stage clinical studies.

## 6. Conclusions and Outlook

The four retinal CNG channelopathies are severe inherited retinopathies leading to either achromatopsia or a retinitis pigmentosa phenotype. Although they are rare disorders, the estimated total number of affected patients is a quarter of a million [42]. Several well-characterized small and large animal models have contributed to a better understanding of the underlying pathomechanisms and are important for preclinical testing of novel therapies based on AAV vectors. Gene therapy programs targeting *CNGA3*- or *CNGB3*-linked ACHM are already in early clinical development but need to demonstrate clinical proof-of-concept in pivotal clinical trials before marketing approval can be granted. Additional programs for CNG-linked RP are expected to follow in the near future to address the remaining gaps in the treatment of *CNGA1*- and *CNGB1*-linked forms of RP.

## Data Availability

No new data were created or analyzed in this study. Data sharing is not applicable to this article.
